# A Virtual Reality Game (The Secret Trail of Moon) for Treating Attention-Deficit/Hyperactivity Disorder: Development and Usability Study

**DOI:** 10.2196/26824

**Published:** 2021-09-01

**Authors:** Maria Rodrigo-Yanguas, Marina Martin-Moratinos, Angela Menendez-Garcia, Carlos Gonzalez-Tardon, Ana Royuela, Hilario Blasco-Fontecilla

**Affiliations:** 1 Department of Psychiatry Instituto de Investigación Sanitaria Puerta de Hierro Segovia de Arana-Puerta de Hierro University Hospital Majadahonda Spain; 2 Autonoma University of Madrid Madrid Spain; 3 Universitat Pompeu Fabra Mataro Spain; 4 Clinical Biostatistics Unit Health Research Institute Puerta de Hierro-Segovia de Arana Center for Biomedical Research in Epidemiology and Public Health Network Madrid Spain; 5 Spain Biomedical Research Networking Center for Mental Health Network (CIBERSAM) Madrid Spain; 6 ITA Mental Health Madrid Spain

**Keywords:** attention-deficit/hyperactivity disorder, chess, virtual reality, serious video game, psychotherapy, cognitive training, usability, new technologies, transfer, randomized controlled trial

## Abstract

**Background:**

Attention-deficit/hyperactivity disorder (ADHD) affects between 4% and 8% of children worldwide. The treatment of choice is multimodal treatment. Multimodal interventions for ADHD may be improved by incorporating new treatments, such as treatment via serious video games. *The Secret Trail of Moon* (TSTM) is a virtual reality serious video game that was designed for cognitive training related to core ADHD symptoms and executive dysfunction.

**Objective:**

We aimed to describe the development and usability of TSTM.

**Methods:**

The usability study included 37 children and adolescents who tested TSTM during the early usability stage (preinclusion) of a randomized controlled clinical trial for testing the effectiveness of TSTM. Chi-square tests were performed to compare patients with ADHD (ADHD combined subtype vs inattentive subtype) and to compare frequent and infrequent video game players in the second study. We used SPSS version 20 for Macintosh (IBM Corporation).

**Results:**

A total of 31/37 (86%) and 30/37 (83%) of participants liked playing TSTM and wanted to continue playing TSTM, respectively. Further, 5/37 (14%) of participants reported that they experienced either perceived dizziness or virtual reality motion sickness. We found no statistically significant differences after comparing the ADHD combined subtype to the inattentive subtype and frequent video game players to infrequent video game players.

**Conclusions:**

Serious video games, such as TSTM, may complement the current multimodal approach for treating ADHD.

**Trial Registration:**

ClinicalTrials.gov NCT04355065; https://clinicaltrials.gov/ct2/show/NCT04355065

## Introduction

### Background

Attention-deficit/hyperactivity disorder (ADHD) is the most common neurodevelopmental disorder of childhood and adolescence; it affects 4% to 8% of children worldwide [1]. Apart from the core ADHD symptoms (inattention, hyperactivity, and impulsivity), patients with ADHD frequently present with poor social skills, problems in planning, and the inability to complete tasks on time [2]. The prognosis of ADHD is complicated by comorbidities, and impairments may intensify during adolescence or adulthood [2,3]. The treatment of ADHD is multimodal and can include the use of medication, psychoeducation, and psychological intervention [4]. Unfortunately, the current multimodal approach for ADHD treatment has some shortcomings [5]. For instance, motivation is critical for people with ADHD, and they sometimes lack the motivation to engage in treatment [6]. Furthermore, psychotherapies can be expensive [7] and have high rates of treatment discontinuation [8,9]. As such, the incorporation of new treatments that promote high levels of motivation may be a good strategy for improving ADHD outcomes and prognoses.

Some recent proposals include the use of board games, such as chess [10]; neurofeedback [11,12]; virtual reality (VR) [13]; or serious video games [14]. All of these new approaches have the potential to keep people with ADHD motivated and engaged during therapy. Indeed, serious video games can be very stimulating and provide immediate reinforcement [15]. In addition, they present some advantages, such as [15] (1) the precise control of variables, (2) easy data collection that allows for the evaluation of a patient's progress, (3) the provision of immediate feedback to the user, and (4) a more attractive presentation (ie, a video game format). It is not surprising that various serious video games have recently been developed to treat ADHD [15-17]. However, it is also important to mention some disadvantages. For instance, a major problem for people with ADHD is their vulnerability to some addictions, particularly the addiction to video games. Children and adolescents with ADHD are more likely to present with internet gaming disorder [18]. Thus, when developing a serious video game for treating ADHD, it is necessary to find a balance between obtaining a good level of user satisfaction and avoiding increasing the risk of becoming addicted to this video game. Independent of the factors that influence addiction in the design of a serious video game, researchers can control the patients who enter into a study, since addiction is linked with adverse childhood experiences [19,20] and game addiction is specifically linked with ADHD severity level [21].

Another problem is the lack of evidence regarding the transfer of improvements and benefits. In other words, it is not known whether improvements in video game performance would translate into improvements in other cognitive tasks in a subject's daily life. For instance, regular chess use has been demonstrated to transfer benefits to the educational domain (eg, by improving mathematics performance) [22]. However, evidence about a potential transfer to the health domain is lacking. The challenge of cognitive training and transfer was addressed by Rabipour and Raz [23]. These authors recommended the potential use of brain training to ameliorate the undesired symptoms of ADHD. They also raised the question of the transfer “of practiced skills to other untrained cognitive domains.” Furthermore, high-quality evidence that supports the massive use of video games to treat ADHD is scarce. Indeed, there is just 1 video game that has recently been approved by the Food and Drug Administration for decreasing the severity of ADHD [24].

The development of any serious video game requires a series of stages [15,25,26]—(1) defining the learning goals (theoretical background and initial design); (2) creating prototypes (proof of concept); and (3) testing usability and clinical effectiveness (in this order). A recent example is *Plan-It Commander*—a serious game that was developed for children with ADHD [15]. They initially defined the learning goals and created a prototype to test the game’s usability and user satisfaction [15]. Afterward, they tested its clinical effectiveness in a randomized controlled trial [16]. They found that girls as well as boys with higher levels of conduct problems were the subgroups that benefited the most from playing the video game [27].

In addition to the development stages reported above, we present the third step (usability) of the development of *The Secret Trail of Moon* (TSTM)—a serious video game that was specifically created to train patients with ADHD and increase various cognitive abilities. This usability study allowed us to (1) obtain initial feedback from patients with ADHD; (2) detect bugs and integrate improvements; and (3) confirm that the use of our VR video game was attractive, was intuitive, and did not generate severe adverse effects in users.

### Deconstructing TSTM: Theoretical Background, Design, Development, and Description

#### Development Stages

**Figure 1:**
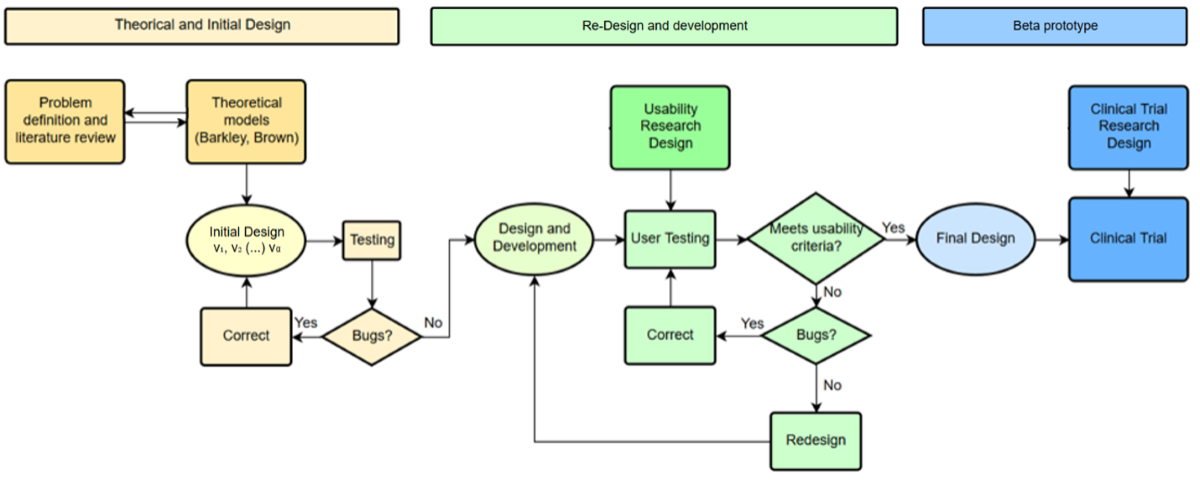
Design and development stages of the user-centered design study.

The development stages of TSTM are shown in Figure 1.

#### Theoretical Background

Information about the first stage of TSTM development can be found elsewhere [28]. The main characteristics of TSTM are summarized in Table 1. These characteristics were influenced by work that was conducted by others [29].

TSTM is the result of interdisciplinary collaboration among health care professionals, researchers, serious video game consultants, and a single educational and therapeutic game development company. This collaboration made it possible to integrate several theoretical models of ADHD with clinical experience, the use of chess and VR by patients with ADHD, and other playful elements of video games that increase players’ motivation for engaging in therapy [30].

TSTM is theoretically driven by (1) both the Barkley [2] and Brown [21] models of ADHD and executive dysfunction and (2) our personal clinical experience of using chess with patients with ADHD [10]. The original Barkley model was based on the work of other researchers, such as Bronowski and Fuster [31,32]. This model focuses mainly on the hyperactive-impulsive ADHD subtype (type 2). The primary premise of the Barkley ADHD model is a deficit in behavioral inhibition. This deficit in inhibition is related to problems in the following four executive neuropsychological functions: (1) working (nonverbal) memory; (2) the self-regulation of affect-motivation and arousal; (3) the internalization of speech (verbal working memory); and (4) reconstitution, which is understood as the ability to manipulate verbal and nonverbal mental representations (behavioral analysis and synthesis) [2].

The Brown ADHD model postulates that the core deficit depends on executive dysfunction. In the Brown model, ADHD is considered to involve 6 major deficits in activation, focus, effort (motivation), emotion, memory, and action. In the Brown model, similar to the Barkley model, executive dysfunction is core to ADHD. However, both models have some differences [21]. The Barkley model focuses on the combined type of ADHD and emphasizes the relevance of behavioral inhibition. In contrast, in the Brown model, behavioral inhibition is just 1 of the 6 defective executive functions [2,21].

The TSTM prototype that was used in this usability study had 5 minigames; each minigame targeted specific cognitive abilities (Table 2) and particularly focused on inhibitory control, selective attention, cognitive flexibility, or processing speed [2,33]. The minigames were embedded into a VR forest context in order to provide a more immersive and motivating experience [28]. Furthermore, as stressed by Rabipour and Raz [23], “regular interaction with nature appears to facilitate improvements in cognitive function and behavioral control.” A minigame was defined as “a small, isolated game within the larger game environment that integrates unique game elements offering tools to improve strategic behavior” [15].

After an initial design of TSTM was produced, we iteratively tested modified versions in order to detect and correct all bugs [34]. Furthermore, we integrated all suggestions that were made by different users who tested the initial version. Accordingly, we included several modifications that allowed us to create a redesigned video game (redesign stage) [34]. We gathered further information on usability criteria, such as effectiveness, efficiency, user satisfaction, and the adverse consequences of use, and fixed some extra bugs. Thanks to this, we constructed the first functional prototype.

In conclusion, TSTM is a serious video game that is defined as either a game that was designed for a primary purpose other than entertainment [35] or a “computer-based [game] designed for training purposes” [36]. We also used gamification [37,38] to introduce chess and to teach the basic rules of chess to participants who did not know how to play the game.

**Table 1:** Brief description of *The Secret Trail of Moon* (TSTM) characteristics (based on Baranowski [30]).

TSTM Characteristics		Description
**General characteristics**		
	Health topics	Attention-deficit/hyperactivity disorder (ADHD) and attention-deficit disorder (ADD)
	Targeted age groups	Individuals aged 12-22 years
	Other targeted group characteristics	Exclusion criteria: epilepsy and dizziness (severe)
	Short description of the game idea	A VR^a^ serious video game aimed at cognitive training related to various cognitive abilities and core ADHD symptoms
	Target players	Individual
	Guiding knowledge, behavior change theory models, or conceptual frameworks	The Thomas Brown model of executive functions and the Barkley Behavioral Inhibition Model
	Intended health behavior changes	Improvements in attention-deficit/hyperactivity disorder symptomatology
	Knowledge elements to be learned	Cognitive abilities
	Behavior change procedures or therapeutic procedures used	Feedback and monitoring, the achievement of goals and planning, the shaping of knowledge, repetition, natural consequences, rewards, and regulation and identity techniques
	Clinical or parental support needed	Clinical support
	Data are shared with parent or clinician	Yes
	Type of game	Adventure and puzzles
**Story**		
	Synopsis	A kid appears suddenly in a cave and is greeted by a curious black fox that talks. While traveling together, they eventually meet a scurrying raccoon, and together they form the MOON^b^ team. Through their adventures in the woods, they will learn about an impending war between two animal factions that want to fill the power vacuum that the King of the Forest—Cernuous—left when he vanished. Wanting to unite all animals again, they set out on a quest to find Cernuous and put an end to the war that threatens the coexistence and nature of the forest itself.
	How the story relates to targeted behavior change	This is a VR adventure experience that is augmented by some specifically designed mechanics. The main goal of the game is to find the King of the Forest throughout several chapters by following the main storyline of the MOON team while resolving problems (game mechanics) in the forest.
**Game components**		
	Player’s game goals and objectives	Cognitive training by using game mechanics
	Rules	Restricted cognitive training (25 minutes per session and per day) and exploring the forest (10 minutes)
	Game mechanics	*Smasher* (minigame for sustained attention and impulse control), *Enigma* (minigame for working memory), *Kuburi* (minigame for visuospatial ability), *Teka Teki* (minigame for planning), and chess (minigame for reasoning)
	Procedures to generalize or transfer outside of the game	Help enhance metacognitive thinking strategies through game play, clinical support, and VR immersion
**Virtual environment**		
	Setting	In the forest, there are ruins of ancient civilizations that praised chess.
**Avatar**		
	Characteristics	The MOON^b^ team consists of the player as well as Movi (raccoon) and One (fox), who will help and accompany the player throughout the game.
	Abilities	Characters will help regulate behavior and be a role model for the player
	Game platform(s) needed to play the game	PlayStation 4 VR (Sony Group Corporation)
	Sensors used	PlayStation 4 VR sensor (Sony Group Corporation)
	Estimated play time	6-8 hours

^a^VR: virtual reality.

^b^MOON: Movi and One.

**Table 2:** Description of each minigame.

Minigame	Target	Description
*Smasher* 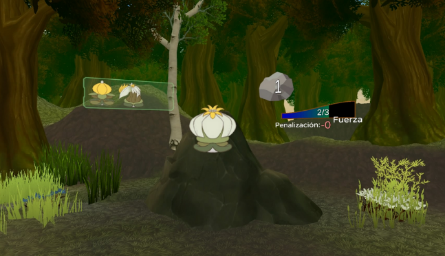	Sustained attention (based on the Brown model of attention-deficit/hyperactivity disorder) Inhibitory control	Within this minigame, participants must break a rock that is blocking their way by following the appropriate set of chess pieces.
*Enigma* 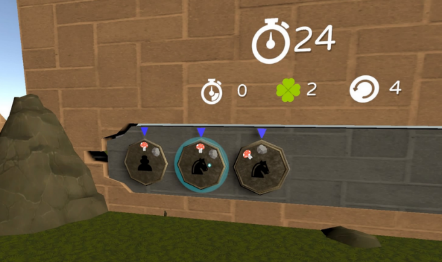	Working memory Cognitive flexibility	Participants must memorize the associations among different elements. Afterward, they must match the association as quickly as possible.
*Kuburi* 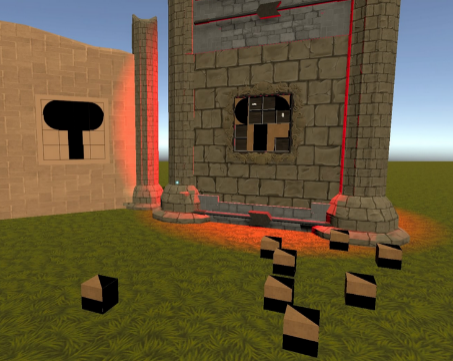	Visuospatial ability According to the classic Baddeley model (1992), the visuospatial agenda is part of working memory. Cognitive flexibility	Participants must create a drawing by using the face and orienting some cubes.
*Teka Teki* 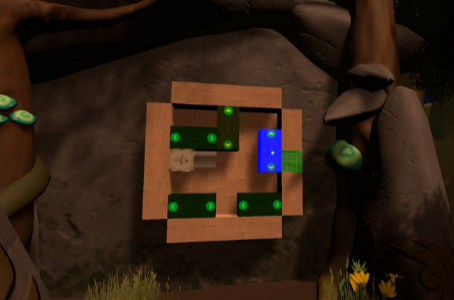	Planning game	Participants can obtain a key if they can help the fox follow her path to the lock. However, the path is obstructed by different blocks. The number of possible movements will decrease, thus increasing the difficulty.
Chess 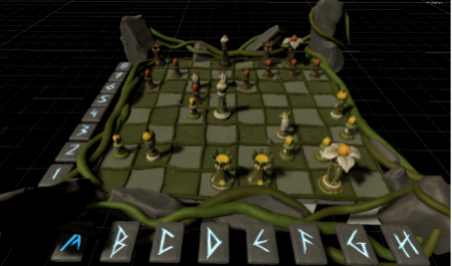	Reasoning Planning Math calculations	Participants must learn the basic rules of chess (ie, the movement of pieces, value of pieces, most relevant moves, etc). The level of difficulty progressively increases.

## Study 1 (Market Study)

### Methods

#### Sample and Procedure

The objective of our market study was to determine whether brain training via a serious video game, such as TSTM, would be of interest to mental health and education professionals. A survey that included the characteristics of TSTM and general questions about the game was designed. We used the Google Forms platform to disseminate Spanish and English versions of the survey through professional networks [39]. A total of 57 people responded, but 1 person was excluded, as he was neither a health worker or an education worker (he was a programmer). The local Committee of Medical Ethics did not require official review for study 1.

#### Statistical Analyses

We only analyzed the percentage of responses. No statistical test was needed.

### Results

The survey was completed by 56 mental health (mostly psychologists and neuropsychologists) and education professionals (teachers, pedagogues, counselors, etc), of which 71% (40/56) either treat and educate or have treated and educated people with ADHD. A total of 91% (51/56) and 87% (49/56) of the professionals thought that a serious video game such as TSTM could be useful and stated that they would use it as a therapy and educational tool, respectively (Table 3).

**Table 3:** Results of our market survey study (study 1).

Factors and questions	Yes, n (%)
Potential use: “Do you think a scientifically validated tool with these features which can help you in ADHD treatment could be useful?”	51 (91)
Practical use: “Would you use it in therapy session?”	49 (87)
Profile adaptation: “Do you think it is relevant to be able to adapt the tool to various ADHD profiles? (Inattentive, hyperactive...)”	54 (96)
Adaptive settings: “Would you like to be able to modify variables according your patient needs? (Distracters, number of elements to memorize...)”	55 (98)
Enjoyable immersion: “Would you like the game to tell a story (script, plot, characters...) in order to facilitate immersion?”	44 (75)

### Discussion

Responses about the characteristics of TSTM helped us to prioritize some development goals Some of the recommendations that were made were in line with literature that stressed the relevance of using attractive graphics or minigame mechanics with an increasing difficulty curve [15,40].

## Study 2 (Usability Study)

### Methods

#### Sample and Procedure

The second study included the first 40 consecutive patients with ADHD who were offered inclusion into a randomized controlled clinical trial (RCT) for testing the effectiveness of either TSTM or web-based chess [41]. This RCT study was a prospective, unicentric, randomized nonequality trial for patients with ADHD. All participants underwent drug titration (up to the optimum drug dose) and were determined to be clinically stable before the baseline evaluation. Patients were randomized into the following three groups: TSTM group (cognitive training via TSTM), the therapeutic chess group (web-based cognitive training via chess), and the control group (patients were called every week, but no cognitive intervention was used) [42]. The allocation ratio was equal in all 3 groups (35 participants per branch).

The patients included in this early usability stage tested the initial version of TSTM between December 6, 2019, and February 22, 2020, at preinclusion. In order to provide all available information before entering into the RCT, patients were offered the opportunity to test TSTM at preinclusion. Of the first 40 patients, 3 were excluded from the statistical analyses either because they did not complete the test or because the data provided were incomplete. The remaining 37 patients tested TSTM and eventually entered the study. However, not all patients were allocated to TSTM group, as randomization took place during the inclusion visit (day 0), and the data presented in this paper were recorded at preinclusion.

The inclusion criteria were (1) an age of 12-22 years, (2) competency in the Spanish language, and (3) written informed consent. The exclusion criterion was epilepsy, since the use of VR is not recommended for individuals with epilepsy, as per the official recommendations of PlayStation VR [43]. Ethical approval was obtained from the local Committee of Medical Ethics at Puerta de Hierro University Hospital – Majadahonda (Madrid, Spain; research project code: PI 187/19; approved on December 1, 2019). Written informed consent was obtained from participants and at least 1 parent.

The materials used for this study included the video game itself (TSTM), VR glasses, PlayStation 4 controllers (Sony Group Corporation), test kit consoles, monitoring screens, and headphones. The VR software runs on a PlayStation 4 (Sony Group Corporation) test device, which allows for the tracking of movement through the camera (eg, position, head movement, speed, etc).

**Figure 2:**
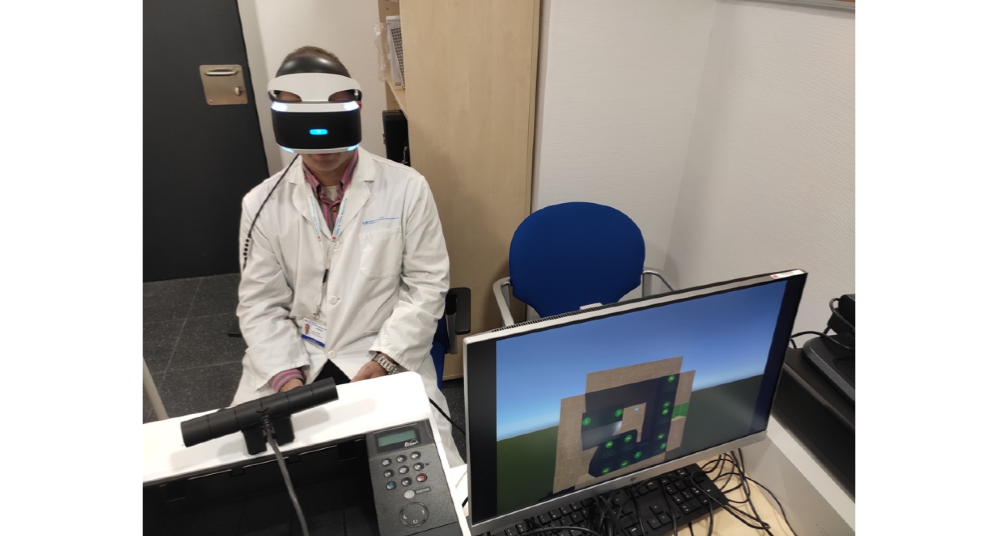
Picture of the principal investigator (HBF) facing *The Secret Trail of Moon*.

Demographics, previous VR experience, and vision problems were noted by using an ad hoc questionnaire. The testing time was always monitored by a professional looking through a screen (Figure 2).

Initially, participants attended a single session in which they tested the TSTM experience. Patients with ADHD were given the opportunity to test a single minigame. After passing the tutorial of the minigame to be tested, a higher level of difficulty was tested. Patients could test each minigame for as long as they liked (ie, only for the duration of that session). After testing a single minigame, patients rated their opinion about the minigame by using an ad hoc questionnaire, which included (1) 5 general questions about the TSTM experience and (2) 2 questions about possible adverse effects. Based on previous studies [44-46], we included information about perceived dizziness and motion sickness, which were rated by using a 3-point Likert scale and 2 questions about patients’ feelings before and after testing TSTM. Standard questionnaires were used as a reference [47] by adapting them to the characteristics of our video game and using language adapted for children. We arbitrarily determined that a user satisfaction rate of 75% would indicate a good level of acceptance by users.

#### Statistical Analyses

Participants were divided into the ADHD combined and inattentive subtypes because some authors consider these subtypes to be different disorders and believe that they should be treated separately. Thus, we feared that the different subtypes of ADHD would impact our usability measures. A descriptive analysis was performed by using the mean proportions (%) of participants’ ratings for categorical variables and means and SDs for numerical variables. Chi-square tests were performed to compare patients with ADHD (ADHD combined subtype vs inattentive subtype) and to compare frequent and infrequent video game players. We used SPSS version 20 for Macintosh (IBM Corporation).

### Results

#### Sociodemographic Characteristics

Table 4 displays the basic demographic and clinical characteristics of participants. The testing time for the video game lasted between 10 and 40 minutes (mean 21.31 minutes, SD 6.77 minutes). Most participants were male (25/37, 68%), were right-handed (29/37, 78%), never repeated a school year (26/37, 70%), and were diagnosed with at least 1 comorbid mental disorder (28/37, 76%). Further, 38% (14/37) of participants wore glasses, 59% (22/37) had previously used VR, and 70% (26/37) played video games regularly. We found no statistically significant differences between the combined and inattentive subtypes of ADHD.

**Table 4:** Demographics of the attention-deficit/hyperactivity disorder (ADHD) and attention-deficit disorder (ADD) subtypes (N=37).

Demographic characteristics			All subtypes (N=37)		Participants with ADHD (combined subtype; 21/37, 57%)		Participants with ADD (inattentive subtype; 16/37, 43%)		*P* value	
Age (years), mean (SD)^a^			13.78 (2.28)		13.38 (2.156)		14.31 (2.387)		.222	
Intelligence quotient, mean (SD)^b^			106.42 (17.91)		104.07 (16.69)		108.76 (19.44)		.525	
**Gender, n (%)**										
	Male	25 (68)		14 (67)		11 (69)		1		
Repeated at least 1 school year, n (%)			11 (30)		7 (33)		4 (25)		.723	
**Handedness, n (%)**										.595
	Right-handed participants	29 (78)		16 (76)		13 (81)				
	Left-handed participants	1 (3)		1 (5)		0 (0)				
	Ambidextrous participants	5 (14)		2 (9)		3 (19)				
Comorbidity with at least 1 mental disorder (yes), n (%)			28 (76)		16 (76)		12 (75)		1	
Wears glasses, n (%)			14 (38)		9 (43)		5 (31)		.432	
Previous use of virtual reality, n (%)			22 (59)		12 (57)		10 (63)		1	
Regularly plays video games, n (%)			26 (70)		16 (76)		10 (63)		.203	

^a^Data were collected from 37 participants.

^b^Data were collected from 26 participants.

#### Usability Results

**Figure 3:**
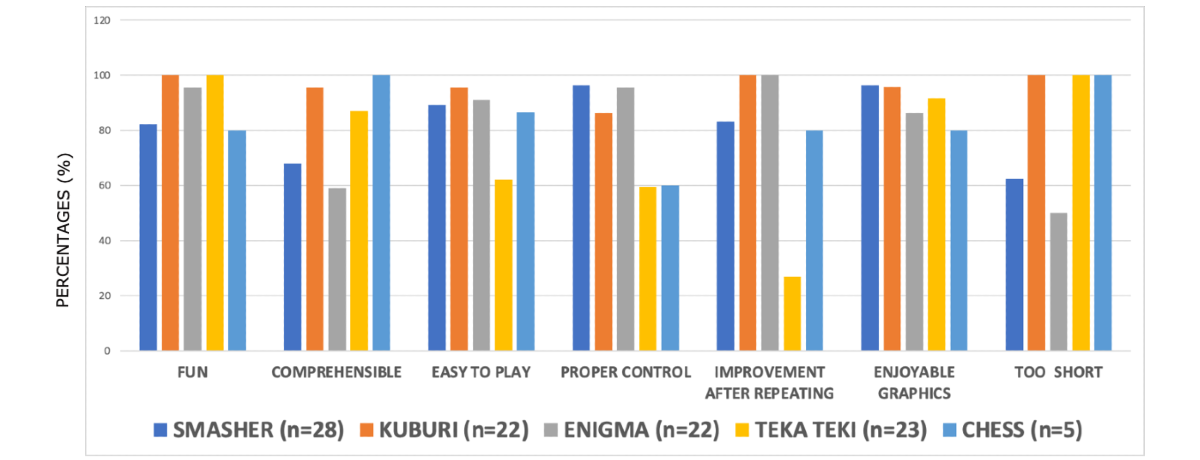
Specific questions about each game’s mechanics (*Smasher, Kuburi, Enigma, Teka Teki*, and *chess*). The questions were as follows: (1) “Was the minigame fun” (factor: fun), (2) “Did you find it easy to understand the instructions” (factor: comprehensible), (3) “Did you find the minigame easy to play” (factor: easy to play), (4) “Did you find the controller easy to use” (factor: proper control), (5) “Do you understand the game better as the level increases” (factor: improvement after repeating), (6) “Did you think visual graphics were beautiful” (factor: enjoyable graphics), and (7) “Did you find it short” (factor: too short)?

Figure 3 displays the proportion of children and adolescents who liked each minigame. All minigames were based on the opinions of the participants, and percentages ranged from 80% (chess: 4/5) to 100% (*Kuburi*: 22/22; *Teka Teki*: 23/23). With regard to the results for comprehensibility, the ease of play, and the ease of control of the PlayStation 4 controller, all proportions surpassed 60% (22/36). All minigames were easy to play, and only *Teka Teki* had an easy-to-play percentage of below 80% (22/36, 61%). *Teka Teki* was the only minigame in which the participants pointed out that it was difficult to achieve improvement after repeating the minigame. We found no statistically significant differences after comparing the ADHD combined subtype to the inattentive subtype and frequent video game players to infrequent video game players.

Table 5 displays the percentages of children and adolescents who had positive opinions, which were based on the two highest scores (“much” or “very much”). With regard to the discomfort of VR glasses and motion sickness, we considered any level of discomfort to be a negative opinion, even if it was just “a little bit” of discomfort. Most children and adolescents provided very positive responses to all questions. Furthermore, 5 out of 36 (13.1%) children and adolescents reported the discomfort of VR glasses and motion sickness, and just 1 (3%) reported this clearly.

**Table 5:** The satisfaction of patients with attention-deficit/hyperactivity disorder after testing *The Secret Trail of Moon* (TSTM).

Factors and questions (responses)	Positive experience (n=36^a^), n (%)
Satisfying experience: “Did you like the experience?” (much or very much)	31 (86)
Desire to repeat: “Would you repeat it?” (much or very much)	30 (83)
Enjoyable graphics: “Did you find it beautiful?” (much or very much)	30 (83)
Enjoyable music and sound: Did you like the music and sound?” (much or very much)	27 (75)
Easy to understand: “Did you find it easy to understand the game?” (much or very much)	28 (78)
Perceived dizziness: “Did you feel dizzy when playing the video game?” (much or very much)	5 (14)
VR motion sickness: “Did you become motion sick while playing the video game?”	5 (14)
Feelings before testing TSTM: “How do you feel?” (good or very good)	26 (73)
Feelings after testing TSTM: “How do you feel?” (good or very good)	36 (100)

^a^Information regarding satisfaction was not recorded for 1 patient out of the 37 included in the second study.

### Discussion

Our study expands on current knowledge concerning the development of serious video games for treating patients with ADHD [15-17,25-28,33,48]. Before serious video games are incorporated into the multimodal treatment of ADHD, they must demonstrate clinical usefulness and have tolerable side effects [15,25,26,28].

Our results suggest that TSTM was fun, was understandable, was easy to play, was intuitive, was easy to master, used enjoyable graphics, and was of adequate duration for most participants. However, there were some interesting differences between each minigame that may suggest that certain components of TSTM need more improvement. The most enjoyable minigames (*Teka Teki* and *Kuburi*) were the most dynamic and interactive minigames. The areas for improvement in *Smasher* appear to be making it a little bit easier to understand and extending the play time. *Kuburi* was the minigame with the highest usability ratings across all parameters. *Enigma* was the minigame that was the most difficult for users to comprehend. Thus, we may have made the graphics too attractive or enhanced minigame mechanics with an increasing difficulty curve [15,40]. *Teka Teki* was the most difficult minigame to play and the only minigame in which participants found it difficult to achieve improvement after repeating the minigame. As such, this minigame may need some improvement, such as using a less stringent difficulty curve and thus preventing a potential decline in initial motivation [15,40]. As for chess, we obtained the opinions of just 5 patients. Therefore, there is too little information to extract any meaningful data on the game.

Compared to a similar study, our study yielded better satisfaction percentages than those reported by the users of *Plan-It Commander* [15]. For instance, compared to the 72% of participants who reported feeling good or very good before testing *Plan-It Commander*, 100% (36/36) of our participants reported feeling good or very good after testing TSTM. Furthermore, our results are comparable to those reported in the *Plan-It Commander* study with regard to players’ motivation to play the game again and their opinions of the game [15]. Finally, a small proportion of participants (5/36, 14%) reported experiencing either perceived dizziness or motion sickness, which was clinically meaningful in just 1 child (1/36, 3%). Wearing glasses was not related to either of the two side effects. Both perceived dizziness and motion sickness are the potential side effects of VR that are the most frequently reported in literature [44-46].

Our usability study presents the following limitations. First, not all of the participants tested the same versions of each minigame during the usability phase, although the changes made were kept at a minimum. We used the preinclusion period of the RCT to fix the bugs detected by participants, and we made some improvements based on their ongoing suggestions in an iterative, continuous way. Thus, the data reported in this paper were the data collected during the preinclusion period. However, after the preinclusion period, all included participants in the RCT eventually tested the same TSTM version. Second, our questionnaire was not validated but was based on questionnaires that were used in similar studies [15]. Finally, we based TSTM development on 2 predominant theoretical models of ADHD. However, there are other ADHD models that integrate cognitive and affective science data and may be interesting to consider when constructing a therapeutic video game for treating ADHD [30,49].

## Conclusion and Future Directions

Serious video games and VR are new technologies that can be used as therapeutic tools for the treatment of mental disorders [24,50]. Compared to traditional treatments, serious video games have many advantages [48,50], such as helping individuals maintain their commitment to therapy [2,13,14]. Furthermore, VR has already proven its therapeutic utility for some mental disorders [47,51,52], but such evidence for ADHD is limited [53]. Moreover, there is an increasing number of studies that have reported encouraging results about the use of serious video games and gamified versions of different tests for treating and diagnosing ADHD populations, respectively [15-17,54-56]. Thus, in a recent clinical trial that included 857 children with ADHD, the researchers reported that the patients who were randomized to a serious video game (Akili interactive) intervention group improved more than those who were randomized to the digital control intervention group and experienced fewer adverse events [24]. In another study that compared a serious game intervention (*Plan-It Commander*) group to a treatment-as-usual crossover group (the intervention was used as an adjunct treatment for children with ADHD), the 10-week serious game intervention proved to be a more effective strategy [16]. However, the creation, development, and empirical testing of a serious video game are not easy tasks [29,57]; the game must be proven to be safe, and its use is not easy to implement in real settings.

TSTM—a VR serious video game that was designed for patients with ADHD—was fun, was intuitive, and displayed a favorable profile of side effects that were in line with those reported in literature [44-46]. Additionally, TSTM may have the potential to be used as an add-on cognitive training tool for medically treated patients with ADHD.

## Abbreviations

ADHD: attention-deficit/hyperactivity disorder
RCT: randomized controlled clinical trial
TSTM: The Secret Trail of Moon
VR: virtual reality
